# The IGNITE (investigation to guide new insight into translational effectiveness) trial: Protocol for a translational study of an evidenced-based wellness program in fire departments

**DOI:** 10.1186/1748-5908-5-73

**Published:** 2010-10-08

**Authors:** Diane L Elliot, Kuehl S Kerry, Esther L Moe, Carol A DeFrancesco, Linn Goldberg, David P MacKinnon, Jeanne Enders, Kim C Favorite

**Affiliations:** 1 Division of Health Promotion and Sports Medicine; Department of Medicine; 3181 SW Sam Jackson Park Road CR110; Oregon Health & Science University; Portland, Oregon 97239-3098, USA; 2 Department of Psychology; Arizona State University; Tempe, Arizona 85287-1104, USA; 3 School of Business; Portland State University; P.O. Box 751; Portland, Oregon 97207-0751, USA; 4 Northwest Fire Fighter Fitness Foundation; P.O. Box 55262; Shoreline, Washington 98155-0262, USA

## Abstract

**Background:**

Worksites are important locations for interventions to promote health. However, occupational programs with documented efficacy often are not used, and those being implemented have not been studied. The research in this report was funded through the American Reinvestment and Recovery Act Challenge Topic 'Pathways for Translational Research,' to define and prioritize determinants that enable and hinder translation of evidenced-based health interventions in well-defined settings.

**Methods:**

The IGNITE (investigation to guide new insights for translational effectiveness) trial is a prospective cohort study of a worksite wellness and injury reduction program from adoption to final outcomes among 12 fire departments. It will employ a mixed methods strategy to define a translational model. We will assess decision to adopt, installation, use, and outcomes (reach, individual outcomes, and economic effects) using onsite measurements, surveys, focus groups, and key informant interviews. Quantitative data will be used to define the model and conduct mediation analysis of each translational phase. Qualitative data will expand on, challenge, and confirm survey findings and allow a more thorough understanding and convergent validity by overcoming biases in qualitative and quantitative methods used alone.

**Discussion:**

Findings will inform worksite wellness in fire departments. The resultant prioritized influences and model of effective translation can be validated and manipulated in these and other settings to more efficiently move science to service.

## Background

Frequently, there is little relationship between the science supporting an intervention and its adoption, and programs are selected based on availability, opportunity or perceived benefits, rather than solid evidence of effectiveness [1]. Most research on moving evidence-based interventions to practice involves programs to alter providers' care patterns or new curricula introduced to schools. Those translational models may differ from worksite dissemination, where adoption is by an organization and participants are asked to alter their existing personal health behaviors, rather than an organization implementing a new curriculum or technology for use with students or clients. No published study has prospectively assessed the complete translation of a worksite health promotion program.

Understanding worksite health promotion is important, as job settings are natural formats for program delivery. Occupational settings have potential to restructure environments and alter social norms, leading to outcomes that benefit both workers and their employers [2,3]. Despite studies documenting reduced healthcare costs and improved employee productivity, evidenced-based worksite interventions often are not used, and those that are used frequently have not been assessed for effectiveness [4,5].

### Conceptual basis and design rationale

This protocol is designed to establish the characteristics of a theory-based, empirically derived framework for worksite translation. Our model's underpinnings are from three perspectives: review of implementation studies [6]; business/organizational psychology [7,8]; and prior experience in the fire departments obtained during the program's development and efficacy trials.

**Figure 1:**
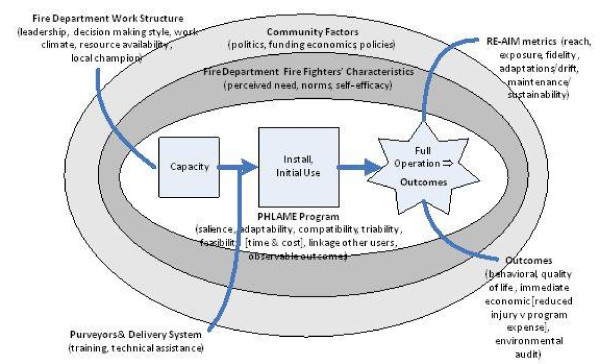
**Framework for Effective Translation**. Modified from Durlak and DuPre [6].

Durlak and DuPre [6] summarized results from more than 500 implementation studies and compared their conclusions and those from two additional reviews. They identified consistent implementation factors related to the setting, the users, the innovation, and its delivery system. Those constructs, along with aspects of an ecological model and organizational analysis, are shown in Figure 1. Final outcomes for our protocol include process evaluation [9], external validity measures of the widely applied RE-AIM framework http://www.re-aim.org, and individual workers' behavioral changes. The framework's sequence of stages provides benchmarks for protocol implementation. In addition, this model will guide the planned mediation analyses.

### PHLAME worksite wellness for firefighters

Despite public perceptions about firefighters being fit, their health profile is comparable to other workers, with many prevalent harmful behaviors: unhealthy diet, lack of regular physical activity, and overweight/obesity [10-12]. Firefighters' episodic intense work, combined with those individual health risks, likely contribute to myocardial infarction being the leading cause of on duty death [13]. In addition, perhaps related to exposure to toxins, their risk of cancer is increased [14,15]. The fire service also is one of the most hazardous occupations, and the rate of work-related injuries is four to eight times greater than that of comparable industries [16]. Prior efforts to mandate health promotion within the fire service largely have been unsuccessful [17].

The PHLAME (promoting healthy lifestyles) wellness/injury reduction program was developed, tested for efficacy, and beta-tested with NIH funding. Its effect sizes were moderate for both diet and physical activity behaviors, and injuries were reduced [18-20]. PHLAME is listed on the Cancer Control P.L.A.N.E.T. evidenced-based website for both promoting healthy nutrition and enhancing physical activity http://cancercontrolplanet.cancer.gov/. However, as with other science-based programs, PHLAME has been used by only a few of the more than 30,000 US fire departments housing more than one million firefighters.

**Figure 2:**
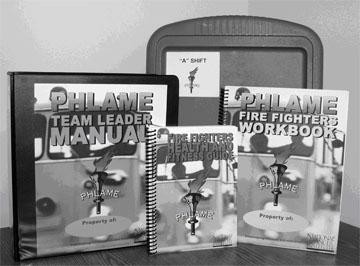
**Curriculum components for the PHLAME program**.

PHLAME's theoretical underpinnings are based on the Health Belief Model [21] and Social Cognitive Theory [22], enhanced by peer effects through a cohesive team work structure [23]. The curriculum is a set of 12, 45-minute interactive sessions, which are completed once per week over approximately four months. The sessions are interactive and based on adult learning principles, emphasizing relevance, problem solving, and application of new abilities [24]. Its team-centered, peer-led format is a natural fit for firefighters' work structure. Typically three stable shifts, composed of four to eight firefighters, staff a fire station, with each shift working 24 hours followed by 48 hours off duty. Accordingly, shifts or work groups can become teams, with sessions inserted into their usual activities. Prior to the first session, one shift member is designated as the team leader, and she/he receives orientation with a training DVD and brief instructional manual. To enhance fidelity and ease of use, the program is explicitly scripted with a team leader manual, elective manual, corresponding workbooks, and an expert resource guide. The materials are stored in the station in a team box between sessions to allow access and provide a visual cue concerning the program (Figure 2).

Non-comparability among businesses and turbulence within and across sites makes many worksites problematic study environments [25]. The fire service has advantages in their hierarchical structure and relatively stable funding base. However, fire departments differ in components such as their size, location, revenue sources, job descriptions, organizational climate, and competing economic demands. Accordingly, the planned cohort design is anticipated to have sufficient variability in key features to establish a theory-based translational model.

### Aims

The goal is to define a model for successful translation by determining the probability of the specified proximal and distal outcomes with different combinations of influential factors/constructs (*e.g*., dimension of departments, purveyors, change agents, and other contextual factors) among a defined population of 12 varied moderately-sized fire departments in Oregon and Washington. Findings from this project will assist worksites/communities in the adoption and effective use of worksite wellness programs; and the translational model can be validated and manipulated in this and other settings to better understand and make translation more efficient.

## Methods

### Study design and phases

This protocol is a prospective cohort observational study [26,27]. The potential predictors and model constructs are theory-based, clearly defined, and feasible to measure, which will increase generalizability and applicability of findings. Data will be gathered in five phases, with attention to the components of the STROBE Statement [28].

### Phase One: Dissemination for awareness

Information about the PHLAME team program and IGNITE study will be sent to all 70 moderately-sized fire departments (40 to 140 career firefighters) in Oregon and Washington. Three individuals per site will be targeted: fire chief, union president and the 'wellness coordinator,' with a personalized letter, informational brochure, and recruitment DVD. The DVD is a three-minute high-impact video production of PHLAME information, program benefits, and participant testimonials. The International Association of Fire Fighters is a strong union, and contacting the union president is an effort to ensure line firefighter representation in the decision to participate. We anticipate fielding contacts and sharing additional information from departments that express interest. From those expressing interest, we will select departments for PHLAME installation based on their commitment and projected ability to involve more than two-thirds of their career firefighters.

### Phase Two: Decision to adopt

**Figure 3:**
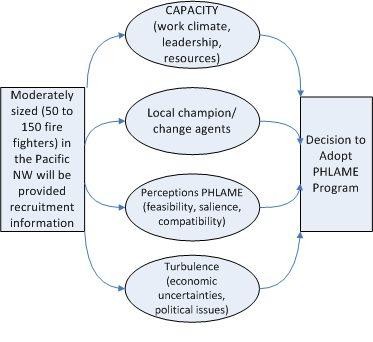
**Decisional Balance Mediation Model**.

Once interested departments are identified, investigators will select 12 sites, after reviewing demographics and contact notes to identify a spectrum of contextual variables with oversampling of sites in minority and lower socioeconomic status (SES) communities. Information about the decision to adopt will be collected during those sites' initial data gathering visits. To better understand the adoption process, we also will collect data from 24 matched non-adopting departments, using phone interviews of those sent the informational packet. For analysis, we will index the adoption decision as both binary yes/no and as a continuous variable combining confidence and self-efficacy, comparable to self-determination theory decision metrics [29]. These data will be used in our mediation analysis of factors contributing to the decision to adopt a worksite wellness program (Figure 3).

### Phase Three: Initial site data and program instillation

Once a department is selected, each site will be assessed over three days (one day per shift), thereby accomplishing data collection for all sites over approximately two months. At each visit, we will obtain consents, distribute/collect surveys, acquire limited physiological data -- body mass index (BMI) and blood pressure -- and conduct focus groups and interviews. We anticipate high participation due to our established credibility from our prior research, demonstrated ability to maintain confidentiality, the camaraderie of firefighters, and convenient onsite data acquisition. With our past firefighter research, participation has been approximately 90 percent [18]. Following data gathering, the site visits will allow in-person orientation of most team leaders. In addition, we can establish plans for follow-up visits and ties for technical support during program use.

### Phase Four: Monitoring program use

A program's initial use may be a particularly critical period. As with any new behavior, system inertia must be overcome, and new activities can feel awkward, potentially resulting in early programmatic failures. This period will be an interval of heightened site observations, and we will continue to record and log any assistance required. The translation literature also suggests that change agents/program champions may have key abilities to influence translation within an organization [30]. Accordingly, we will gather data specifically relating to these key members using observations and the post-program surveys.

We also will have random visits (approximately two per site) to observe sessions and conduct focus group data collection of firefighters and department administrators during the latter weeks of program use. While technical support will be readily available when requested, the PHLAME observation efforts will remain separate from the data collection staff.

### Phase Five: Follow-up data and outcomes

**Figure 4:**
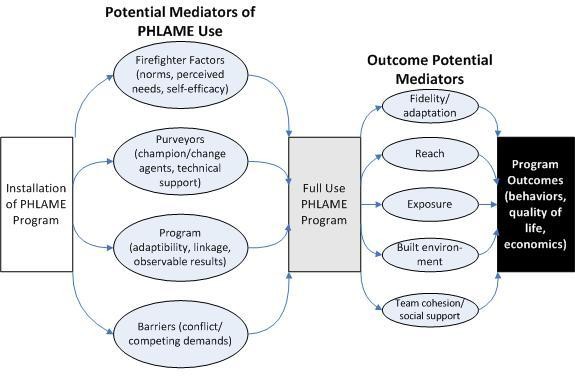
**Translation Mediation Model**.

Approximately six to eight months following a department's PHLAME installation, we will begin a second round of three-day visits, which will repeat the initial data gathering activities. In addition, the follow-up assessments will include information relating to program outcomes (*e.g*., number participating [reach], dose delivered, dose received by participants, and fidelity to the scripted manual/workbook format). Information will be used in this phase's mediation assessment (Figure 4).

### Data collection instruments

The constructs and components shown in Figure 1 will be assessed in data collection. The questionnaires used will have high face validity, with item selection based on empirical evidence, theory, validity/reliability, and relevance. Many of the constructs will have established relevance and reliability from our prior work [18,20]. We anticipate a nine-page instrument, which in our experience is within the response burden tolerance of firefighters. The individual outcome and demographic measures include anthropometric measures (height, weight, calculated BMI), dietary measures (validated National Cancer Institute [NCI] fruit and vegetable screener [31,32]), self-reported physical activity, sleeping (reliable items modified from Division of Sleep Medicine at Brigham and Women's Hospital worker studies), organizational features [33,34], occupational fatigue items [35], quality of life, and additional individual variables (perceived family impact, age, gender, race/ethnicity, years as a firefighter, current position/job, and years to retirement). Economic outcome data will be of two types: self report injury and illness, and intervention costs, not counting research inputs, as recommended by the Panel on Cost Effectiveness in Health and Medicine [36]. A list of the items making up physical activity, exercise support, nutrition knowledge, diet support and quality of life are available at http://www.public.asu.edu/~davidpm/ripl/Phlame.htm.

The focus group and key informant semi-structured interviews will include items that provide additional understanding of model constructs [37]. Open-ended questions to explore emergent themes will be used, with later exploration of relevant domains. The business literature offers findings that will be useful in understanding the antecedents and motivational factors relating to program adoption, including access to resources, proactive personality style, and leadership role efficacy using established, reliable constructs [38]. Information from the human resource literature will be used to assess perceived organizational impact, social consensus/pressure, decision-making style, readiness to change, and climate (clarity of mission and goals, cohesiveness, stress, and openness to change).

### Data analysis

#### Quantitative data

In general this analysis will use SPSS (SPSS, Chicago, IL) and M-Plus for structural equation modeling (SEM). Survey instrument assessment will begin by confirming predicted item constructs, augmented with exploratory factor analysis, to establish reliable summary scales with maximum internal consistency. Having reduced the survey items to a manageable number of robust constructs, the relation of variables in the translational model will be evaluated. For continuous outcomes, structural equation modeling will be used to evaluate relations among variables using model fit indices. For binary or ordinal outcomes, each construct's contribution to predicting group states for outcomes will be conducted using logistic regression analysis. Cross-sectional and longitudinal models will be developed and model fit indices calculated.

Mediation can address how an intervention achieves its effects [39-41], and it will be used to explain relations between the purported mediators and the outcomes as predicted in Figures 3 and 4. The goal of mediation analysis is to determine which aspects of an intervention are contributing to change, and it defines means for their modification and improvement.

#### Qualitative data

Interviews and focus groups will be audiotaped and transcribed. Transcripts will be read for emerging themes, and then imported into atlas.ti software for review and coding into categorical data in the dimensions of interest. Those groupings will begin with our theoretical survey constructs, and those propositions will be refined and expanded as data emerges. The software tabulates frequencies of events or categories, allows chronological assembly to establish patterns and arraying data using different analytic strategies/graphic displays. Findings will be refined with validity checks, including establishing redundancy, respondent validation, and clear exposition of methods.

#### Triangulation of quantitative and qualitative data

The quantitative and qualitative data paradigms will be combined, with adjustment for the particular study phase [42,43]. For the initial decision to adopt, the individual survey items will inform the mediation analysis, and additional decisional aspects explored in the qualitative data. The latter translational sequences will use the combined survey data, with qualitative findings used to expand on, challenge, and confirm survey findings. Combining both analysis types will provide a richer understanding, confirmatory convergent validity, completeness, and confidence of data by overcoming biases in either method used alone.

Gathering qualitative findings also will allow developing case studies [44]. Often in the business community, information is shared as descriptive cases, and for selected departments, we will create case studies, describing the sequence and identified factors relating to translation. Case studies are intense investigations of specific instances, and generally are evaluated for their usefulness and whether the descriptions are contextually complete. We anticipate that these case studies may be useful when sharing findings with the community of firefighters and fire department decision makers.

### Study power

Analysis of cross-sectional survey data for outcomes will have sufficient power to detect small effects, with adjustment of the multilevel structure of the data. For the more comprehensive covariance structure models, power depends on several factors, such as the number of parameters, effect sizes, levels of analysis, and measurement model. Based on rule of thumb ratios of sample size to parameters and Monte Carlo simulation of latent variable models, this study has a power of approximately 0.4 for a small effect, 0.7 for a moderately small effect (halfway between small and medium), and 0.97 for medium effects. In general, the sample size is sufficient to estimate moderately sized latent variable covariance structure models that include constructs at both the individual level and departmental level. We acknowledge that model performance is likely to be overestimated in a single dataset, and internal validation techniques will be used to assess for and correct that possibility [45,46].

Calculating power for the mediation analyses is based on newer techniques that incorporate resampling methods and the distribution of the product. Assuming a small intraclass correlation, we will have 0.8 power to detect moderate effect size mediation relations. Power to detect moderator effects is slightly less and will require at least medium effect sizes for most potential moderators.

Sample size for qualitative data will be based on the criteria of representative and collecting information to saturation, so that additional interview/focus groups do not add to emerging data. However, our intent is to gather data from all participants at each site.

### Potential study challenges

Several challenges may occur during the protocol, and plans have been made to prevent and overcome those potential issues. Our protocol is dependent on enrolling departments willing to allow time for the program and data collection, and recruitment is set against a context of fire departments often facing declining funding due to a reduced property taxes. However, especially in the Pacific Northwest, PHLAME has recognition as an effective program, and we believe that the potential of acquiring the program at no cost, along with effective promotional material, will result in adequate participation among the 70 potential departments. If needed, we can add personal contact and extend recruitment to other departments.

A second issue is the geographic dispersion of the 12 departments within Oregon and Washington, which will necessitate traveling and three-day stays to those sites. Our protocol is budgeted to accommodate those needs, and many potential locations are within one day's travel from our base, which is centrally located within the two state areas. An explicit manual of operations and training data collectors will provide consistency in those efforts.

The fire service is a unique occupation, which could limit model generalizability [47]. For example, unlike most worksites, many fire stations have exercise equipment, so that efforts to combine individual and environmental components in a worksite wellness program are less of an issue. As we analyze data, findings will be used to understand our settings' ecology (policies, organizational issues, community and societal issues), and their potential nonlinear influences on translation.

Finally, the process of studying these departments and our visits to gather data and monitor progress would not be present if a department was purchasing PHLAME for independent use. The original description of altered behaviors because of being studied was in a worksite setting, the Hawthorne Plant of the Western Electric Company in Cicero, Illinois [48,49]. We will try to minimize that effect and monitor for it as we assess outcomes, *e.g*., obtaining permission for random visits and asking sites whether they would have completed all the sessions if we were not coming to monitor their progress.

## Discussion

The critical importance of translation is well recognized [50], and findings from this protocol will add to the understanding of worksite health promotion. In a review of translation, Sussman *et al. *[51] identified two important general objectives for translational research, both of which will be achieved in this proposal. First, as recommended, experts from different academic fields and community partners are collaborating to bring perspectives and new insights from their disciplines. Second, findings will provide a toolbox of metrics, instrumental variables, and a framework for translation that can be validated and manipulated in this and other settings.

An established translational model would have immediate benefits for the 30,000 US fire departments as improved worksite safety and wellness will: enhance firefighters' health; reduce costs of injury, illness, and overtime; and allow community funds to be redirected to other jobs and services. This protocol has the potential to define a model for translation and identify the constructs that mediate its stages, from adoption and instillation to full use and behavioral/economic outcomes. Extending a translational roadmap for worksite wellness to other settings could improve health, reduce insurance costs and provide economic stimulus for both employers and workers.

### Ethical aspects

The Institutional Review Board of the Oregon Health & Science University approved the study in August of 2009. Interviews and focus group transcripts are anonymous. After the research assistant(s) who collected the data listens to and reviews transcripts for accuracy, names are removed and those transcripts are only identified by site. Individual surveys and measurements are confidential with a secure code book maintained by the investigator and data manager Participating departments will be provided summative information about their site and de-identified summary data concerning other departments.

## Competing interests

PHLAME is a program on the Cancer Control P.L.A.N.E.T. http://cancercontrolplanet.cancer.gov/ site for research-tested programs, and it is distributed through the Center for Health Promotion Research at Oregon Health & Science University (OHSU). OHSU and Elliot, Goldberg, and Kuehl have a financial interest from the commercial sale of technologies used in this research. This potential conflict of interest has been reviewed and managed by the OHSU Conflict of Interest in Research Committee.

## Authors' contributions

DLE is Principal Investigator on the project and prepared the initial draft of this manuscript. DLE, KSK, ELM, CAD, and LG formulated the study protocol and contributed to drafting the manuscript. DPM assisted in protocol development was instrumental in the quantitative assessment components; JE assisted with a perspective from organizational psychology; and KCF provided a community partner aspect. All authors read and approved the final manuscript.
